# Editorial: Advancements in marine-derived proteins: enhancing nutritional and functional properties

**DOI:** 10.3389/fnut.2025.1720718

**Published:** 2025-11-04

**Authors:** Haisheng Lin, Duanquan Lin, Lei Du, Lester Geonzon, Le Chang Sun

**Affiliations:** ^1^Guangdong Provincial Key Laboratory of Aquatic Product Processing and Safety, College of Food Science and Technology, Guangdong Ocean University, Zhanjiang, China; ^2^College of Ocean Food and Biological Engineering, Jimei University, Xiamen, China; ^3^State Key Laboratory of Bioreactor Engineering, Department of Food Science and Engineering, School of Biotechnology, East China University of Science and Technology, Shanghai, China; ^4^Institute of Life and Environmental Sciences, University of Tsukuba, Tsukuba, Ibaraki, Japan

**Keywords:** marine proteins, protein functionalities, bioactive peptides, nutrition, by-product valorization, enzymatic hydrolysis, digestomics, nanocarriers

## Introduction

The growing global demand for sustainable and nutrient-dense protein sources has accelerated researches into marine-derived proteins, particularly those recovered from aquaculture and fisheries side streams. This Research Topic explores innovative strategies to extract, modify and characterize proteins and protein hydrolysates from under-utilized marine biomass, aiming to enhance their nutritional, functional, and bioactive properties. The seven articles in this Research Topic highlight diverse approaches-from enzymatic hydrolysis and membrane fractionation to ultrasonication and *in-silico* screening-that collectively advance our understanding of how to transform low-value marine by-products into high-value ingredients for food, feed, nutraceutical and biomedical applications.

## Turning waste into worth: re-engineering fish processing by-products for valorization

Fish processing by-products represent an abundant reservoir of high-quality proteins whose recovery and valorization are pivotal for sustainable resource utilization and circular bioeconomy advancement. Two back-to-back studies on Italian sea-bream/sea-bass trims showed how a single pre-processing technology propagates through the entire valorization chain. Jenssen, Sone et al. and Jenssen, Matic et al. first revealed that industrial dehydration, while reducing moisture and transport cost, locked the protein matrix into a less hydrolysable state, yielding ~3 kDa peptides with 30–40% lower antioxidant activity and diminished ACE-inhibitory potency.t. Yet the same groups also demonstrated that a membrane (molecular weight cutoff = 3 kDa) separation can redeem the handicap. The permeate fraction, lighter in color and rich in low-molecular-weight peptides, accelerated *in-vitro* wound closure by >40% and retained hepatoprotective activity. Complementarily, the companion paper maps the full bifurcation: raw trims yield higher dry-matter hydrolysis efficiencies and smaller peptides (<2,600 g mol^−1^), whereas dehydrated trims deliver bulkier hydrolysates better suited for techno-functional roles—emulsifying, foaming, oil-binding—once recombined with the retentate. Together, the duo delivers a practical blueprint: dehydrate for logistics if needed, but always pair with size-selective fractionation to recover bioactive permeates and functional retentates, thereby converting perishable side streams into dual-purpose ingredients for nutraceuticals and clean-label foods. Similarly, Cropotova et al. show that post-hydrolysis ultrasonication combined with *Aeromonas proteolytica aminopeptidase* can shift the molecular-weight distribution of Atlantic mackerel hydrolysates toward smaller, more soluble peptides without increasing bitterness, which proofs that “green” physical/enzymatic finishing steps can fine-tune both nutrition and sensorial quality.

## Structural diversity drives application specificity

Turning shells into high-performance materials begins with recognizing that “crustacean” is not a single feedstock but a spectrum of nano-architectures. Yang et al. remind us that even the supporting polymer matrix-chitin-displays source-dependent nano-architectures. Antarctic krill, white shrimp and crayfish chitins differ in crystallinity (78–87%), thermal stability and surface porosity, dictating downstream suitability for biomedical scaffolds vs. food-grade films. Their systematic comparison provides a materials-science rationale for diverting crustacean shells away from landfill and toward biodegradable packaging, reinforcing the principle that understanding intrinsic macromolecular context is prerequisite to any rational up-cycling strategy.

## From hydrolysate to active peptide: *in silico* acceleration

The discovery of novel bioactive peptides is a key driver for valorizing aquatic proteins, and the development of rapid, high-throughput screening strategies has become a central research priority. Qiao et al. applied virtual screening to abalone viscera hydrolysates and identified four <1 kDa peptides that inhibit HMG-CoA reductase more effectively than atorvastatin (IC_50_ equivalent) in hyper-lipidaemic Hep-G2 cells. While Lin et al. converted *Chlamys nobilis* muscle into EHCA, achieving 35 % α-glucosidase inhibition and DPPH scavenging; in mice it enhanced glucose tolerance and hepatic SOD while lowering MDA. Two peptides (TDADHKF and KLNSTTEKLEE, IC_50_ = 144–137 μM) out-competed acarbose through stable hydrogen-bond interactions, providing scalable marine leads for glycaemic control. These studies showcase how LC-MS/MS *de-novo* sequencing, molecular docking and 300-ns MD simulations can compress years of empirical screening into weeks, yet still demand rigorous in-cell and *in-vivo* validation.

## Health modulation beyond classical nutrition

Li et al. employed RNA-seq to dissect tropomyosin-driven allergic responses in murine jejunum and Caco-2/RBL-2H3 models, revealing PI3K/Akt/NF-κB as the central axis by which shrimp tropomyosin compromises tight-junction integrity and amplifies histamine release. Their findings not only inform hypo-allergenic processing strategies (e.g., controlled hydrolysis to ablate epitopes) but also exemplify how marine proteins can serve as probes to map fundamental gut-immune interactions.

## Processing quality and endogenous enzyme interplay

Cropotova et al. again highlight that endogenous proteases and physical treatments (ultrasound) interact synergistically to modulate peptide size, free amino-acid profiles and color—critical parameters for industrial acceptance. Real-time monitoring via NIR or Raman spectroscopy, coupled with adaptive process control, is proposed as the next step toward batch-to-batch consistency of marine protein ingredients.

Collectively, these studies emphasize several key advancements:

Process optimization: Enzymatic hydrolysis conditions, pre-treatment methods, and post-hydrolysis modifications (ultrasonication, membrane fractionation, aminopeptidase finishing) critically influence hydrolysate characteristics and must be co-designed within a circular biorefinery framework.

Functionality and bioactivity: Marine protein hydrolysates exhibit promising emulsifying, antioxidant, wound-healing, anti-diabetic and hypo-cholesterolaemic properties that can be enhanced through targeted processing and *in-silico* design.

Sustainability: Efficient conversion of side streams (heads, viscera, shells) into proteins, peptides and chitin supports circular-economy principles and reduces environmental impact.

Future research should focus on (i) pilot-scale validation of energy and mass balances, (ii) systematic allergenicity mapping and mitigation strategies, and (iii) long-term human intervention studies to translate *in-vitro* promise into dietary guidelines. Integrating multi-omics datasets with machine-learning models will further accelerate the discovery of sequence-defined peptides and optimize process parameters in real time.

This Research Topic provides a comprehensive roadmap for transforming the “blue granary” into a resilient source of sustainable, health-promoting protein ingredients, offering actionable insights for scientists, industry stakeholders and policymakers committed to a sustainable food future.

